# Engraftment of human mesenchymal stem cells in a severely immunodeficient mouse

**DOI:** 10.1186/s41232-024-00353-2

**Published:** 2024-09-26

**Authors:** Yuko Kato, Yusuke Ohno, Ryoji Ito, Takeshi Taketani, Yumi Matsuzaki, Satoru Miyagi

**Affiliations:** 1 Department of Biochemistry, Faculty of Medicine, Shimane University, 89-1 Enya, Izumo City, Shimane 693-8501 Japan; 2 Human Disease Model Laboratory, Central Institute for Experimental Medicine and Life Science (CIEM), 3-25-12 Tonomachi, Kawasaki-Ku, Kawasaki, 210-0821 Japan; 3 Department of Pediatrics, Faculty of Medicine, Shimane University, 89-1 Enya, Izumo City, Shimane 693-8501 Japan; 4 Department of Life Science, Faculty of Medicine, Shimane University, 89-1 Enya, Izumo City, Shimane 693-8501 Japan; 5 PuREC Co., Ltd., 89-1 Enya, Izumo City, 693-8501 Japan

**Keywords:** Mesenchymal stem cells, Mesenchymal stromal cells, Xenograft model, NOG, Transplantation, Regenerative medicine

## Abstract

The transplantation of human mesenchymal stromal/stem cells (hMSCs) has potential as a curative and permanent therapy for congenital skeletal diseases. However, the self-renewal and differentiation capacities of hMSCs markedly vary. Therefore, cell proliferation and trilineage differentiation capacities were tested in vitro to characterize hMSCs before their clinical use. However, it remains unclear whether the ability of hMSCs in vitro accurately predicts that in living animals. The xenograft model is an alternative method for validating clinical MSCs. Nevertheless, the protocol still needs refinement, and it has yet to be established whether hMSCs, which are expanded in culture for clinical use, retain the ability to engraft and differentiate into adipogenic, osteogenic, and chondrogenic lineage cells in transplantation settings. In the present study, to establish a robust xenograft model of MSCs, we examined the delivery routes of hMSCs and the immunological state of recipients. The intra-arterial injection of hMSCs into X-ray-irradiated (IR) NOG, a severely immunodeficient mouse, achieved the highest engraftment but failed to sustain long-term engraftment. We demonstrated that graft cells localized to a collagenase-released fraction (CR), in which endogenous colony-forming cells reside. We also showed that Pdgfrα^+^Sca1^+^ MSCs (PαS), which reside in the CR fraction, resisted IR. These results show that our protocol enables hMSCs to fulfill a high level of engraftment in mouse bone marrow in the short term. In contrast, long-term reconstitution was restricted, at least partially, because of IR-resistant endogenous MSCs.

## Background

Colony-forming unit-fibroblasts (CFU-F) and the ability to differentiate into adipogenic, osteogenic, and chondrogenic progeny (trilineage differentiation capacity) are hallmarks of cultured mesenchymal stromal/stem cells (MSCs) [1, 2]. Therefore, human MCCs (hMSCs) are attractive cell sources for regenerative medicine for congenital skeletal diseases characterized by bone structure fragility [3, 4]. Historically, MSCs have been enriched based on adherence to plastic plates and expansion in cultures [5, 6]. The properties of MSCs were characterized in vitro using cell surface marker expression, cell proliferation assays, and differentiation assays [1, 2]. In mice, a prospective isolation protocol using FACS with monoclonal antibodies against cell surface marker(s) and reporter gene(s) enables us to isolate various MSC fractions directly from mouse bone marrow (BM) [7]. In addition, the development of genetic tools, e.g., the *Cre-LoxP* system, can assess mouse MSCs (mMSCs) dynamics in an animal context and reveal their self-renewal and differentiation capacities under physiological and pathological conditions [8, 9]. For example, BM reticular cells expressing high levels of CXCL12 are called CXCL12-abundant reticular (CAR) cells [10], which also express the Leptin receptor and are called Lepr^+^ MSCs. CAR/Lepr^+^ MSCs exhibit self-renewal and multilineage differentiation capacities in vitro [8]*.* Seike et al. showed that the *Ebf3-CreERT2; Rosa26-tdTomato* transgene labeled CAR/Lepr^+^ MSCs with tdTomato upon treatment with tamoxifen and labeled CAR/Lepr^+^ MSCs were maintained as MSCs for more than one year, demonstrating the lifelong self-renewal capacity of CAR/Lepr^+^ MSCs [11]. This methodology is robust but not applicable to cultured MSCs, including hMSCs. Therefore, in vitro assays are still the gold standard for validating the ability of hMSCs [1, 2]. It remains unclear whether cultured hMSCs show self-renewal capacity in the clinical transplantation setting or in living animals. In other words, it has yet to be established whether cultured hMSCs engraft and permanently contribute to tissue regeneration in patients. The transplantation assay is an alternative approach to validate the self-renewal capacity of stem cells [12, 13]. However, the efficiency of MSC engraftment may be increased, even in syngeneic transplantation [14]. Zhou et al. demonstrated that CAR/Lepr^+^ MSCs formed bone, cartilage, and adipose four weeks after an intrafemoral injection. However, graft cells only accounted for 1% of CAR/Lepr^+^ MSCs in recipient femurs, indicating inefficient self-renewal activity [8]. In most studies, the efficacy of hMSC transplantation has been ambiguous because the authors only described the biological efficacy of hMSCs, not the exact efficiency of engraftment [15]. Furthermore, few studies quantified the content of MSCs in a specific organ in recipients and reported poor engraftment [16]. However, the degree of engraftment is an essential factor affecting the clinical outcome of regenerative medicine.

We previously demonstrated that LNGFR^+^THY-1^+^ cells in human BM were highly enriched for MSCs that clonally grow. Among these clones, fast-growing clones are called rapidly expanding clones (RECs) and also show trilineage differentiation capacity [17].

The present study aimed to establish an efficient xenograft mouse model of hMSCs using RECs as an MSC model. We examined some steps of the MSC transplantation and evaluation protocol for recipient mice. Our optimized protocol allows hMSCs to efficiently contribute to the BM stromal fraction for one week; however, this contribution decreases with time. An analysis of the stromal fractions of recipients demonstrated that X-ray irradiation (IR)-resistant MSCs might restrict the long-term reconstitution of MSCs.

## Main text

### Comparison of intracaudal arterial (CA) and intravenous (IV) injections

**Fig. 1:**
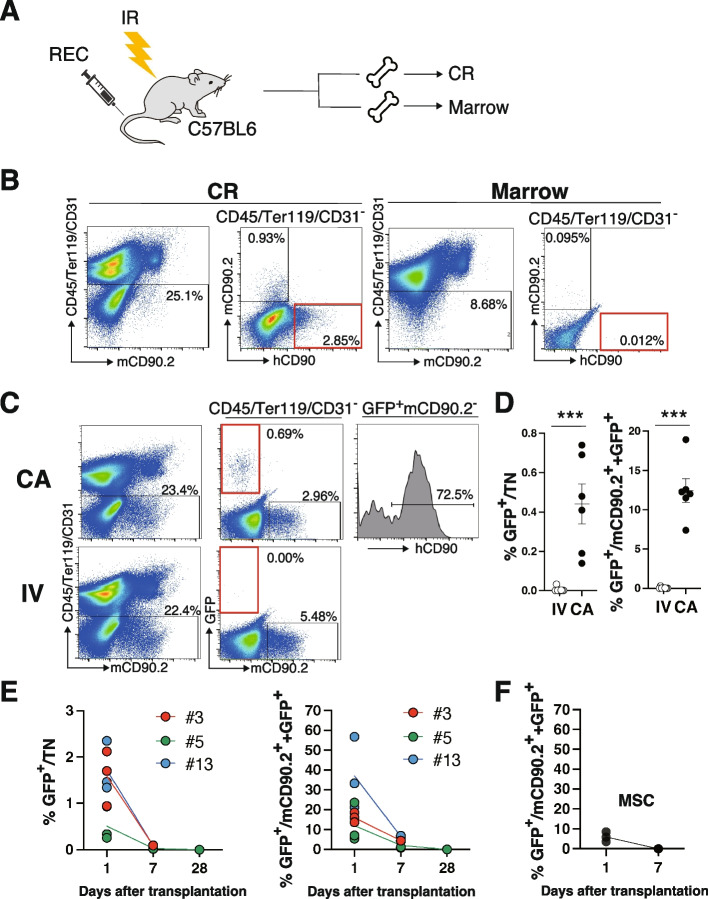
The CA injection improves MSC engraftment. **A** Experimental design. **B** One day after GFP + REC transplantation into C57BL6, CR (Left panel) or marrow (Right panel) fractions were prepared from a pair of tibia and femur and stained with the indicated antibodies for the FCM analysis. **C** Representative FCM profiles of the CR fraction from C57BL6 recipients transplanted with GFP + RECs through the CA or IV injection. **D** The frequencies of GFP + cells in the TN (Left panel) or stromal fraction (Right panel) were calculated from the FCM profile in (**C**). **E** Time course changes in GFP positivity in the TN or stromal fraction from C57BL6 transplanted with GFP-labeled REC (clones #3, 5, or 13) (**E**) or MSCs (**F**) are shown. Data are shown as the mean ± SEM. *P < .05; **P < .01; ***P < .005; ****P < .0005; *****P < .00005 by the Student’s *t*-test

We investigated the localization of graft cells in long bones because the anatomical localization of BM-derived MSCs is specific to each MSC subtype [18, 19]. For example, CAR/Lepr^+^ MSCs are part of reticular cells and form a cellular network in the marrow. On the other hand, Pdgfra^+^Sca1^+^ MSCs (PαS) cells reside in the arterial perivascular area near the inner surface of cortical and trabecular bones [20]. Therefore, PαS cells are released from minced bone pieces through collagenase treatment (collagenase-released cell fraction; CR), while LepR^+^ MSCs are isolated from flushed marrow [21]. Four million RECs were IV injected into C57BL6 mice preconditioned with lethal IR. Seventeen hours after transplantation (Day 1), CR was prepared from a pair of tibia and femur, and BM was prepared from the other pair for a flow cytometry analysis (FCM). (Fig. 1A). RECs marked by human CD90 (hCD90) expression were merely detectable in CD45^−^Ter119^−^CD31^−^ non-hematopoietic and epithelial cells (Triple negative fraction; TN) from the marrow whereas hCD90^+^ cells were observed in the CR fraction (Fig. 1B). We note that hCD90 was not an ideal marker for RECs just after IR because hCD90^dim^ cells were present in the CR fraction from IR mice. The presence of these cells affected our ability to evaluate engraftment quantitively. Therefore, RECs labeled with GFP were used in transplantation experiments hereafter.

An IV injection is widespread as a systemic injection in MSC and hematopoietic stem cell (HSC) research and clinics [15, 22]. It initially results in the accumulation of cells in the lungs, then in the livers, spleen, and kidneys, and, to a lesser extent, other organs, including BM. Therefore, an IV injection of MSCs may cause pulmonary infarction, which is the most frequent complication associated with MSC transplantation in clinical settings. Conversely, CA injection initially delivers cells to the hind limb and then systemically to peripheral organs [22]. Therefore, CA injection is assumed to decrease the risk of pulmonary embolism and lead to a higher engraftment rate especially in hind limb BM. We transplanted GFP-labeled RECs by an IV or CA injection to compare engraftment efficiencies and conducted FCM analysis on Day 1. The CA injection resulted in a significantly higher frequency of GFP^+^ cells in the TN and stromal fractions (mCD90.2^+^  + GFP^+^) than the IV injection (Fig. 1C and D). Furthermore, most graft cells expressed hCD90 in the mouse BM microenvironment after transplantation (Fig. 1C). We also noted that respiratory condition worsened immediately after IV injection of RECs, leading to death in 10–15% of recipients. The cause might be a pulmonary embolism. We then investigated whether RECs retained long-term reconstitution activity. We injected GFP-labeled REC clones (#3, #5, or #13) by CA and tested engraftment on Days 1 and 7 (clones #3 and #5) or Days 1, 7, and 28 (clone #13). On Day 1, the frequencies of GFP^+^ cells in the TN fraction from recipients injected with clones #3, #5, and #13 were 1.89 ± 0.35, 0.51 ± 0.21, and 1.72 ± 0.32%, respectively. GFP^+^ cells were not detectable on Day 7 and 28 (Fig. 1E). The frequency of GFP^+^ cells was markedly higher in the stromal fraction (mCD90.2^+^  + GFP^+^) than in the TN fraction on Day 1. The chimerism of clones #3, #5, and #13 were 16.1 ± 1.37, 12.1 ± 5.78, and 37.1 ± 10.45% on Day 1, respectively (Fig. 1E). GFP^+^ cells were present in the stromal fraction on Day 7, but disappeared by Day 28. We also demonstrated that MSCs established by the conventional protocol showed similar kinetics to REC clones (Fig. 1F).

### Immunosuppression improves the engraftment of hMSCs

**Fig. 2:**
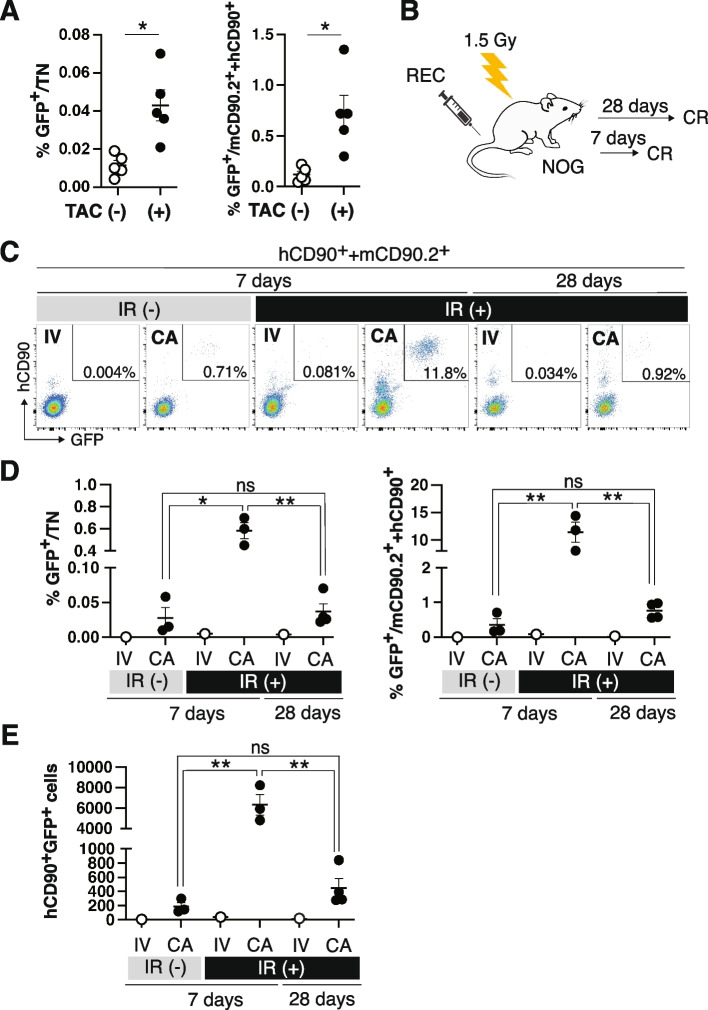
Immunosuppression improves MSC engraftment. **A** Frequencies of GFP^+^ cells in the TN (Left panel) or stromal fraction (Right panel) from mice transplanted with GFP-labeled RECs in the presence or absence of TAC on Day 7. **B** Experimental design. **C** GFP-labeled RECs were transplanted into NOG mice with or without semi-lethal IR through the CA or IV injection. Recipients were analyzed on Days 7 and 28. **D** The frequencies of GFP^+^ cells in the TN (Left panel) or CD90^+^ stromal fraction (Right panel) were calculated from the FCM profile in (**C**). **E** Number of GFP^+^ in a pair of tibia and femur. Data are shown as the mean ± SEM. *P < .05; **P < .01; ***P < .005; ****P < .0005; *****P < .00005 by the Student’s *t*-test

**Fig. 3:**
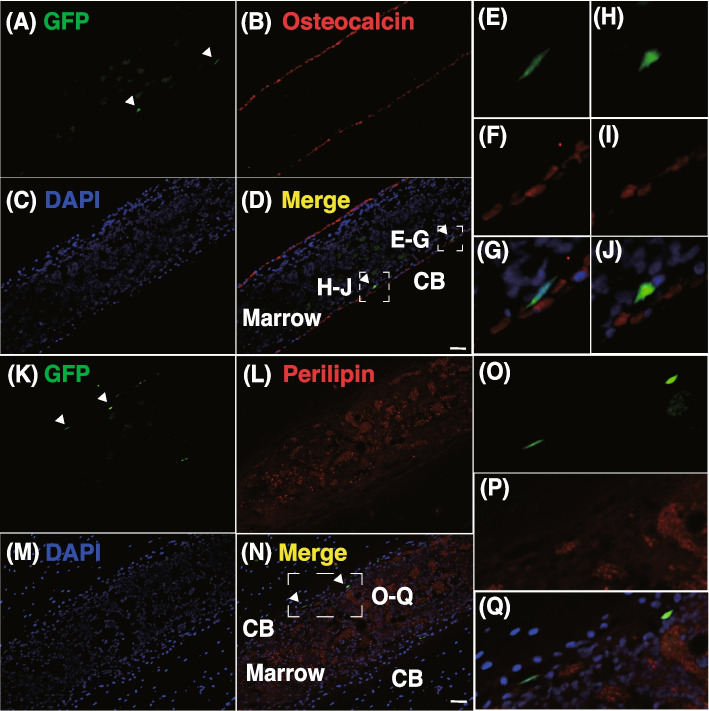
Intra-bone marrow localization of RECs. Coimmunostaining of GFP and Osteocalcin (**A-J**) or GFP and Perilipin (**K-Q**) on the femur sections from the recipient seven days after transplantation. **A**, **K** GFP, (**B**) Osteocalcin, (**C**, **M**) DAPI, (**D**, **N**) Merged images, (**L**) Perilipin. **E-J, O-Q** Magnification images. Arrowheads indicate the GFP-expressing cells. Scale bar, 50 mm; CB, cortical bone

We examined the effects of immunosuppression in the recipient on transplantation outcomes. To achieve this, we treated C57BL6 mice with the immunosuppressor tacrolimus hydrate (TAC) every other day from the day before the CA injection [23]. The FCM analysis on Day 7 revealed that the TAC treatment increased GFP positivity in the TN and CD90^+^ stromal fractions (mCD90.2^+^  + hCD90^+^) on Day 7 (Fig. 2A), demonstrating that immunosuppression significantly improved the engraftment of hMSCs considered to have low immunogenicity. We then conducted MSC transplantation utilizing extremely severe combined immunodeficient NOG (NOD/Shi-scid, IL-2Rγnull) mice, which were established by combining NOD/scid mice and IL-2 receptor-γ chain knockout mice [24]. We transplanted GFP-labeled RECs by an IV or CA injection into NOG mice with or without IR at a semi-lethal dose (Fig. 2B). We tested the frequency of hCD90^+^GFP^+^ cells in the CD90 fraction on Days 7 and 28. As shown in Fig. 2C, the CA injection resulted in a higher percentage of chimerism than the IV injection, and an apparent hCD90^+^GFP^+^ cell population was detected, even in non-IR recipients. Grafted cells comprised approximately 10% of the CD90 fraction in CA-injected preconditioned recipients seven days after transplantation. The percentage of chimerism was markedly higher in NOG mice than in C57BL6 mice (compare Fig. 1E and Fig. 2D), indicating that immune rejection is one factor that restricts the engraftment of hMSCs. However, it decreased to approximately 1% in the CD90 fraction on Day 28. The absolute numbers of GFP^+^ cells were 6,318 ± 1,007 and 499 ± 133 in CA-injected preconditioned recipients 7 and 28 days after transplantation, respectively. These results show that hMSCs failed to engraft long-term, even in preconditioned NOG mice (Fig. 2E). We also noted that the most engrafted cells expressed hCD90. Then, we conducted an immunostaining on the femur sections to test whether the engrafted RECs differentiate into osteoblasts and adipocytes in mice. The GFP^+^ cells resided in the endosteal region. However, these cells did not express the osteoblast marker, Osteocalcin (Fig. 3A-J). We also tested the expression of GFP and adipocyte marker, Perilipin, and found that their expression is mutually exclusive (Fig. 3K-Q). These results and the fact that most engrafted cells retained CD90 expression revealed that RECs did not show overt differentiation in living animals in seven days after transplantation.

### IR-resistant endogenous MSCs restrict the engraftment of hMSCs

**Fig. 4:**
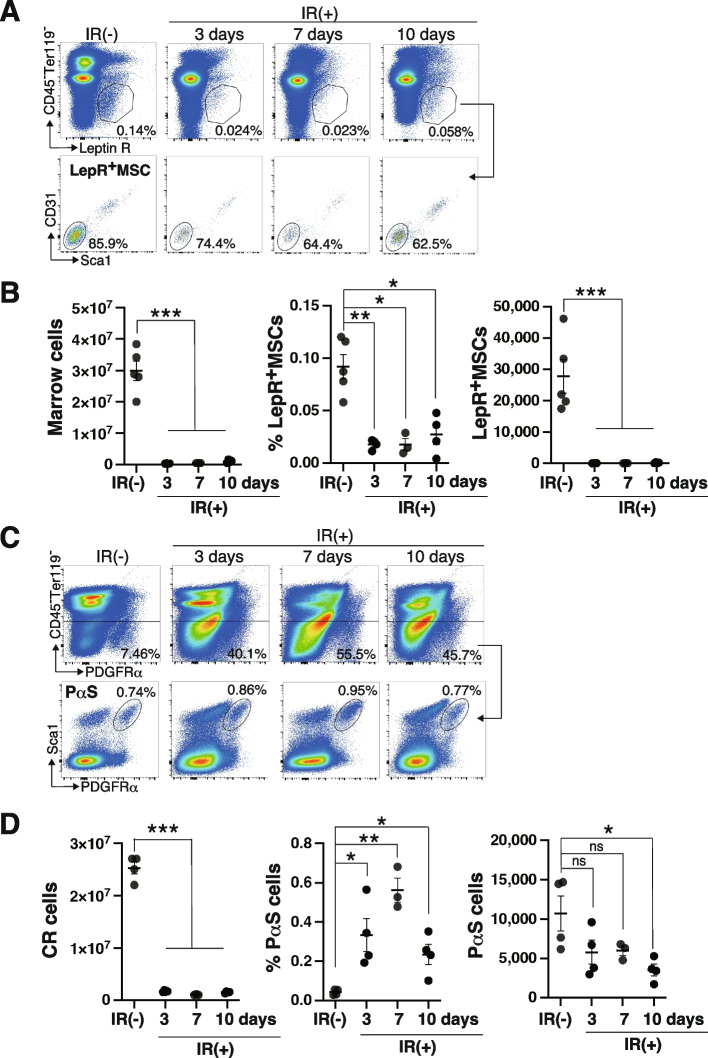
PαS cells show IR resistance. C57BL6 mice were X-ray irradiated and the frequencies of PαS and CAR/LepR^+^ MSCs were examined on Days 0, 3, 7, and 10. **A**, **B** Representative FCM profiles of CAR/LepR^+^ MSCs (**A**) and the cellularity of the marrow fraction, frequencies, and absolute number of CAR/LepR^+^ MSCs (**B**) are shown. **C**, **D** Representative FCM profiles of PαS cells (**C**) and the cellularity of the CR fraction, frequencies, and absolute number of PαS cells (**B**) are shown. Data are shown as the mean ± SEM. *P < .05; **P < .01; ***P < .005; ****P < .0005; *****P < .00005 by the Student’s *t*-test

We investigated why hMSCs failed to engraft over one week in NOG mice efficiently. To achieve this, we examined the responses of endogenous mMSCs to IR. The absolute number of marrow cells markedly decreased after IR and remained low for up to 10 days after lethal IR in C57BL6 mice. Similarly, the frequencies of CAR/Lepr^+^ MSCs decreased to approximately 10% of the non-IR control three days after IR, as shown before [25], and did not recover during our observation period (Fig. 4A and B). In contrast, lethal IR had a minimal impact on PαS cells. It decreased the absolute number of PαS cells only by 50%, as reported before [20]. We note a significant increase in %PαS in the IR ( +) group attributed to the reduction of hematopoietic cells by IR (Fig. 4C and D). Our previous study showed that PαS cells are mainly found in CR fraction [21]. Histologically, PαS cells reside in the endosteal region like engrafted hMSCs [20]. These results suggest that IR-resistant PαS cells restrict the long-term engraftment of hMSCs, even though the molecular mechanism is still elusive. Generally, cultured MSCs fail to engraft into normal BM in the transplantation setting [26]. Horwitz et al. showed that MSCs engraft into the BM of infant patients with osteogenesis imperfecta (OI) caused by *COL1A1 or COL1A2* mutation [27, 28]. The stem cell factor (SCF) regulates the pool size of HSCs and hematopoietic progenitor cells in the mouse hematopoietic system. HSCs compete with proximal hematopoietic progenitor cells for membrane-bound SCF (mSCF) on MSCs and endothelial cells [29] since their abundance is limited [30]. Thus, it is worth speculating that endogenous hMSCs compete with their progenies for the niche or niche factor in a normal situation. This competition would not occur in OI patients since they lack the cells to compete with MCSs due to impaired osteogenesis. The lack of competition allows exogenous hMSC to graft efficiently in OI patients. Similarly, IR-resistant PαS cells or their progenies may compete with exogenous hMSCs and limit the long-term engraftment of hMSCs. The localization of engrafted RECs in the femur (Fig. 3A-D) is similar to that of PαS cells [20], implying the direct competition between PαS cells and RECs in the endosteal region.

However, IR-resistant MSCs may not be the only factor limiting the engraftment of hMSCs. In the xenograft model of hHSCs, some mouse cytokines have been reported to not function on human cells and fail to support the engraftment and differentiation of hHSCs. Hence, humanizing cytokines significantly improves xenograft outcomes [31, 32]. Therefore, cytokine/growth factor cross-reactivity between mice and humans may also explain the limited engraftment of hMSCs. If this is the case, what cytokine/growth factor (s) are the targets of humanization to improve hMSC engraftment? Several growth factors (PDGFs, TGFs, NGF, FGF2, and EGF) play important roles in MSC biology in vitro and in vivo. A comparison of the amino acid sequences of these factors from human and mouse origins revealed that EGF, PDGF-B, and NGF showed less than 90% identity, while the others showed more than 95%. Among these three factors, PDGF-B was of interest because Yin et al. reported that enforced expression of human PDGFB in hMSCs promotes their proliferation in vitro and survival and expansion in the transplantation setting [33]. Therefore, the *PDGFB* gene is one of the candidates for humanization to improve the xenograft model of hMSCs. It is interesting to test if the humanization of PDGFB alone enables hMSCs to engraft long-term in mice because PDGFB acts synergistically with other growth factors, e.g., VEGF, HGF, or EGF, on MSC proliferation and migration [34].

In this study, we have systematically refined the delivery routes of hMSCs, the preconditioning protocol, and the immunological state of recipients and found that the CA injection of hMSCs into X-ray-irradiated NOG leads to efficient engraftment. Although most hMSCs do not survive beyond a month in the recipients, our xenograft model is still a valuable tool to decipher the molecular mechanisms of immunomodulation and hematopoietic support by hMSCs since hMSCs fulfill their beneficial effects on acute GVHD or allo-HSCT just or soon after transplantation in the mouse model.

## Conclusions

In the present study, we considered the conditions of the hMSC transplantation model. We demonstrated that the CA injection was efficient, resulting in approximately 10% chimerism in the CD90 stromal fraction of the NOG mouse. However, engraftment was not permanent, and graft cells did not survive for one month. The hMSC xenograft model still needs improvement by refining the preconditioning protocol and humanizing soluble factor(s).

## Methods

### Cultures of MSCs and RECs

BM-derived MSCs and RECs were purchased from Lonza (Basel, Switzerland) and PuREC Co., Ltd. (Izumo, Japan), respectively. RECs and MSCs were cultured in DMEM Low Glucose with L-Glutamine (FUJIFILM) supplemented with 20% fetal bovine serum (Hyclone), 1% penicillin–streptomycin (FUJIFILM), 10 mM HEPES (FUJIFILM), and 10 ng/mL basic fibroblast growth factor (bFGF, FUJIFILM). The medium was changed every 2–3 days. MSCs and RECs from passage 4 were infected for GFP marking with the retrovirus vector, *pMYs-IG* (Cell Biolabs, San Diego, USA). Forty-eight hours after transduction, GFP-expressing cells were sorted and expanded for transplantation experiments.

### Mice

NOG mice (NOD.Cg-*Prkdc*^*scid*^*Il2rg*^*tm1Sug*^*/*ShiJic) were purchased from the Central Institute for Experimental Medicine and Life Science (CIEM) (Kanagawa, Japan). C57BL/6 J mice were purchased from Charles River Laboratories Japan, Inc. (Kanagawa, Japan). All animal experiments were performed under our institutional guidelines for the use of laboratory animals and were approved by the Review Board for Animal Experiments of Shimane University (approval ID: IZ5-19).

### Transplantation

Before transplantation, C57BL/6 J and NOG mice were IR at 12 and 1.5 Gy, respectively. Four million RECs or MSCs suspended in 0.2 ml of REC culture media without bFGF were injected into the dorsal vein or caudal artery. In the experiment with lethal IR, unfractionated 10^5^ BM cells were injected together with RECs or MSCs as rescue cells. TAC (FK506, FUKIFILM) was dissolved in PBS at 0.5 mg/ml, and 0.1 ml was injected into mice intraperitoneally every second day from the day before transplantation.

### Cell preparation and flow cytometry

CR and BM fractions were prepared for flow cytometry from a pair of tibia and femur. Cells were stained with fluorochrome-conjugated antibodies recognizing the following antigens: mouse Cd45 (30-F11), mouse Ter119 (TER-119), mouse Pdgfrα (APA5), mouse Sca1 (D7), mouse Cd31 (MEC13.3), mouse leptin receptor (goat polyclonal antibody), mouse Cd90.2, and human CD90 (5E10). Antibodies were purchased from eBioScience, BioLegend, TONBO, and R&D Systems. We also stained cells with 1 μg/mL propidium iodide (Sigma-Aldrich) to eliminate dead cells. Flow cytometry analyses were conducted on CytoFLEX (Beckman Coulter Life Sciences), while cell sorting was performed on MoFlo XDP (Beckman Coulter Life Sciences).

### Immunostaining

The femur and tibia were fixed, decalcified, and embedded in paraffin. After deparaffinization and antigen retrieval with citrate buffer, sections were incubated with anti-osteocalcin (Rabbit polyclonal antibody; Takara Bio) or perilipin A antibody (Rabbit polyclonal antibody; Sigma-Aldrich) with anti-GFP antibody (Chicken IgY; Aves Labs). Antibodies were detected with appropriate Alexa Fluor dye-conjugated secondary antibodies (Thermo Fisher Scientific). Sections were counterstained with 4′,6-diamidino-2-phenylindole (DAPI).

### Statistical analysis

Statistical analyses were performed using Graph Pad Prism version 9. The significance of differences was measured by the Student’s *t*-test. Data are shown as the mean ± SEM. Significance was taken at values of *p less than 0.05, ** *p* less than 0.01, *** *p* less than 0.005, **** *p* less than 0.0005, and ***** *p* less than 0.00005.

## Data Availability

Data that support the results of the present study are provided in this manuscript. Materials are available from the corresponding author, SM, upon reasonable request.

## Abbreviations

FACS: Fluorescence-activated cell sorting
EBF3: Early B-cell factor 3
CXCL12: C-X-C motif chemokine ligand 12
ERT2: Estrogen receptor T2
PDGF: Platelet-derived growth factor
TGF: Transforming growth factor
NGF: Nerve growth factor
EGF: Epidermal growth factor
HEPES: 2-[4-(2-Hydroxyethyl) piperazin-1-yl] ethanesulfonic acid
